# Neisseria meningitidis Serogroup C Causing Primary Arthritis in a Child: Case Report: Erratum

**DOI:** 10.1097/MD.0000000000016858

**Published:** 2019-08-09

In the article, “Neisseria meningitidis Serogroup C Causing Primary Arthritis in a Child: Case Report”,^[1]^ which appeared in Volume 95, Issue 5 of *Medicine,* the first four lines of Table 1 are being removed as they were found to not be serogroup C and one study was added. The publication year, age of patients and joints involved were also corrected.

**Table 1:**
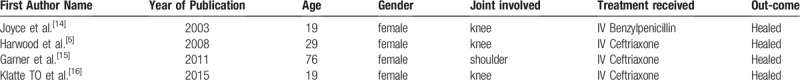
Reported cases of primary arthritis with Neisseria meningitidis serogroup C.

The following references were removed from the article.

13. Joice M, Laing A, Mullet H. Isolated septic arthritis: meningococcal infection. J R Soc Med 2003; 237–238.
14. Christiansen JC. Primary meningococcal arthritis caused by *Neisseria meningitidis*. One of the many manifestations of meningococcal disease. *Ugeskr Laeger* 1995; 157:3909–3910.
15. Cartolano GL, Le Lostec Z, Chéron M, et al Primary meningococcal arthritis of the knee: contribution of synovial liquid culture in blood-culture vial. *Rev Méd Interne* 2001; 22:75–78.
16. Giamarellos-Bourboulis EJ, Grecka P, Petrikkos G, et al Primary meningococcal arthritis: a case report and review. *Clin Exp Rheumatol* 2002; 20:553–554.
